# The early molecular epidemiology of the swine-origin A/H1N1 human influenza pandemic

**DOI:** 10.1371/currents.RRN1003

**Published:** 2009-08-31

**Authors:** Andrew Rambaut, Edward Holmes

**Affiliations:** ^*^The Institute of Evolutionary Biology, University of Edinburgh, Edinburgh & The Fogarty International Center, NIH and ^†^The Pennsylvania State University & The Fogarty International Center, NIH

## Abstract

Swine-origin pandemic human influenza A virus (H1N1pdm) has spread rapidly around the world since its initial documentation in April 2009. Here we have updated initial estimates of the rate of molecular evolution and estimates of the time of origin of this virus in the human population using the large number of viral sequences made available as part of the public health response to this global pandemic. Currently sampled H1N1pdm sequences share a most recent common ancestor in the first 7 weeks of 2009 with the implication that the virus was transmitting cryptically for up to 3 months prior to recognition. A phylogenetic reconstruction of the data shows that the virus has been circling the globe extensively with multiple introductions into most geographical areas.

## Introduction

H1N1pdm (also referred to as S-OIV) is a newly emergent human influenza A virus that is closely related to a number of currently circulating pig viruses in the 'classic North American' and 'Eurasian' swine influenza virus lineages[1] [2]. Since the first reports of the virus in humans in April 2009, H1N1pdm has spread to 168 countries and overseas territories, with >177,000 reported cases[3]. To reveal the early molecular epidemiology of the H1N1pdm, particularly its spatial patterning and evolutionary dynamics, we performed an evolutionary analysis on available genome sequence data sampled globally. The aim of our study was to provide updated information on estimates of the rate of evolutionary change in H1N1pdm, its date of origin, and its growth rate in human populations.

## Methods

H1N1pdm sequences were collated from the NCBI Influenza Database on 6th August 2009. These sequences were then filtered to produce a data set that met the following criteria; that the exact date (day) of collection was given, that both of the hemagglutinin (HA) and neuraminidase (NA) gene sequences were available (any other sequenced genes were also included), and that they had been isolated from humans. This resulted in a data set of 377 isolates. In a previous application of these approaches to sequences collected early in the outbreak[4], it was possible to trace epidemiologically-linked clusters and correct for this sampling bias explicitly by picking one isolate from each cluster. Given the large number of cases globally, this is no longer possible. Therefore, to reduce any effect of over-sampling of epidemiologically-linked isolates (such as those from New York State, USA), a further filtering was applied where, for each day of isolation, only one virus from a given location (country and state) was retained; this resulted in a total of 242 isolates. This final set included isolates from 23 different countries with the majority (118) coming from the USA.

These sequences were then analysed using BEAST v1.5.0[5], an analysis package that uses a Bayesian Markov chain Monte Carlo (MCMC) approach to sample time-structured evolutionary trees from their joint posterior probability distribution derived from a combination of molecular evolutionary and population genetic models. The data were analysed under an exponential-growth coalescent model as a prior on the tree, the HKY+\begin{equation*}\Gamma \end{equation*} model of nucleotide substitution and a relaxed molecular clock[6]. 4 independent runs of 10 million steps were peformed, compared for convergence and combined less a 10% burnin from each.

## Results & Discussion

The results of the Bayesian MCMC analysis are summarized in **Table 1**. The analysis of this much larger sample of H1N1pdm virus confirms that the date of the most recent common ancestor (MRCA) of circulating human lineages pre-dates the first sampled case by approximately 2 months. Assuming that a single cross-species transfer from pigs to humans gave rise to all the sampled H1N1pdm diversity, the date of the MRCA is a lower limit of how recently this event occurred. The estimate presented here represents a considerable increase in precision over that published previously [2] concomitant with the increased period of sampling and greater number of viruses in this study (we estimate a credible interval spanning approximately the first 7 weeks of 2009). In sum, this means that there was a period of up to about 3 months where the virus was circulating in humans prior to initial characterization (cryptic transmission). 

The substitution rate for H1N1pdm is significantly higher than that estimated by Smith *et al.* [2] for each of the genomic segments for a panel of related swine viruses (at ~3 x 10^-3^ substitutions/site/year). This higher rate in H1N1pdm was noted by Smith *et al.*, and as an explanation it was suggested that the rapid epidemic spread and short timescale had resulted in a proportion of mildly-deleterious mutations being maintained in the population. It will be possible to test this hypothesis when H1N1pdm sequences are sampled over a longer time-period.

**Table 1** **. **Marginal posterior estimates of model parameters.

**Table:** 

	Rate of molecular evolution (x10^-3^ subs/site/year)	Date of most recent common ancestor	Exponential growth rate	Doubling time (days)
mean estimate and Bayesian credible interval	5.02 (4.17, 5.95)	27-Jan-2009 (30-Dec-2008, 22-Feb-2009)	14.92 (10.44, 19.61)	17.0 (12.9, 24.2)

Figure 1 shows the maximum clade credibility tree (the tree sampled from the MCMC with the highest product of individual clade probabilities). This tree is intended as a representative tree from the posterior sample; however, the ages of each node in the tree are set to the mean across the entire sample. 

**Figure:**
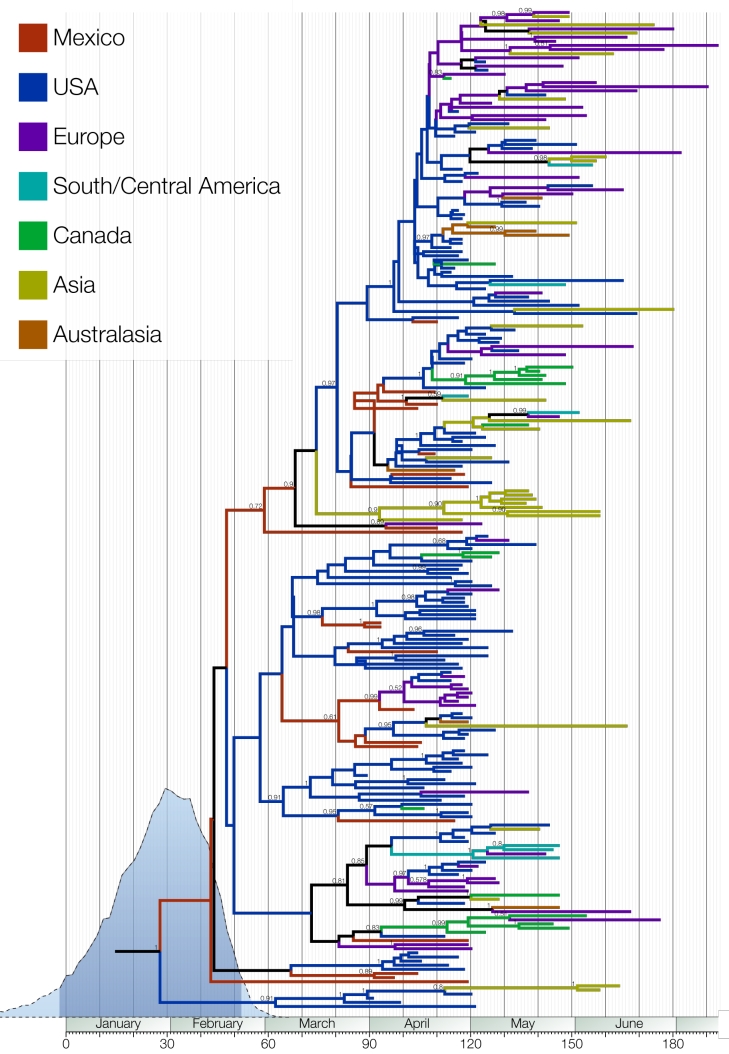
**Figure 1. **The maximum clade credibility (MCC) tree of H1N1pdm. In blue is the marginal posterior probability density of the time of the most recent common ancestors of the sampled lineages (95% credible interval shown by darker blue). Clades are labelled with their posterior probability where greater than 0.5. Lineages are coloured using a parsimonious reconstruction based on the locations of the sampled viruses.

PDF version with isolate names

The color-coded branches highlight the rapid spatial diffusion of H1N1pdm, which multiple entries into countries from Asia and Europe. Such a spatial mixing is typical for influenza A virus [7] and suggests that H1N1pdm exhibits similar spatial dynamics to those of seasonal influenza. It is also notable that multiple lineages of H1N1pdm are circulating in a single geographical region (with the United States a notable example), providing the raw material for intra-subtype reassortment.

The population growth rates and epidemic doubling times inferred here are similar to those estimated previously for H1N1pdm [4] and suggest that the virus will continue to spread globally. However, it is difficult to compare these coalescent-based estimates of epidemiological dynamics with those of seasonal influenza viruses, as multiple introductions into any locality in any season [8] [9] mean that equivalent point-source outbreaks are rarely observed.

Acknowledgments

Thanks to Gytis Dudas for help with collating the sequence data.

Funding information

AR was funded by The Royal Society of London and the Interdisciplinary Centre for Human and Avian Influenza Research (ICHAIR). ECH was funded by NIH grant R01 GM080533. Both AR and ECH thank the Fogarty International Center, NIH, for continued support.

Competing interests

The authors have declared that no competing interests exist.
